# Psychosocial Difficulties in Preschool-Age Children with Beckwith–Wiedemann Syndrome: An Exploratory Study

**DOI:** 10.3390/children9040551

**Published:** 2022-04-13

**Authors:** Niccolò Butti, Annalisa Castagna, Rosario Montirosso

**Affiliations:** 1 0–3 Center for the at-Risk Infant, Scientific Institute, IRCCS Eugenio Medea, Bosisio Parini, 23842 Lecco, Italy; niccolo.butti@lanostrafamiglia.it (N.B.); annalisa.castagna@lanostrafamiglia.it (A.C.); 2 PhD Program in Neural and Cognitive Sciences, Department of Life Sciences, University of Trieste, 34127 Trieste, Italy

**Keywords:** Beckwith–Wiedemann syndrome, emotional-behavioral problems, psychosocial difficulties, psychomotor development, preschool-age children, pediatric chronic illness, rare diseases

## Abstract

Beckwith–Wiedemann syndrome (BWS) is a rare overgrowth disease and is not usually associated with intellectual delay. Living with a chronic illness condition such as BWS, however, might affect emotional-behavioral functioning and psychosocial development. To investigate this issue, parents of 30 children with BWS between 1.5 and 6 years old compiled standardized questionnaires assessing the presence of emotional-behavioral and developmental problems. The group mean scores in each scale of behavioral problems fell within the average range. Nevertheless, 23% of the sample presented scores beyond the risk threshold for social withdrawal. As regards psychomotor development, a lower mean score was reliable in the social domain compared to other developmental scales, and in the gross-motor compared to fine-motor functions. Moreover, scores in the at-risk band were reliable in almost half of the children for social development. Notably, older age was overall associated with higher emotional-behavioral and developmental difficulties, while no other socio-demographic or clinical variables accounted for the scores obtained in the questionnaires. These findings ask for a wider consideration by health and educational professionals of the psychosocial functioning of children with BWS, so as to early detect at-risk conditions and eventually promote adequate interventions.

## 1. Introduction

First described in the 1960s by the parallel work of Bruce Beckwith and Hans Rudolf Wiedemann, Beckwith–Wiedemann syndrome (BWS) is an overgrowth disorder, with an estimated prevalence of 1 in 10,500 newborns [1]. The clinical manifestation is very varied and often includes macroglossia, abdominal wall defects, lateralized overgrowth, enlarged abdominal organs and a heightened risk of developing embryonal tumors. Despite the diagnosis of BWS mainly relying on physician’s clinical assessment and a new scoring system that has been proposed [2], more than three out of four cases of BWS can be ascribed to altered expression of imprinted genes in two functionally independent domains of the chromosome 11p15.5. In detail, approximately 60% of BWS patients present altered expression of the growth suppressor gene *CDKN1C*, mostly due to loss of methylation of the *KCNQ1OT1*:transcriptional start site differentially methylated region (DMR) (also known as IC2) on the maternal allele of the centromeric domain. Less frequent causes are known to be a gain in methylation in the *H19/IGF2*:intergenic differentially methylated region (also known as IC1), associated with increased expression in the growth promoter gene *IGF2* on the paternal allele of the telomeric domain and Uniparental Paternal Disomy (UPD) of 11p15.5 [3].

The complex clinical picture and the presence of different (epi)genetic variants have led research to focus on medical and etiopathogenic aspects of the syndrome [4], while the psychosocial consequences of BWS are not yet fully investigated. The few previous studies documented that risk conditions often presented by children with BWS, such as hypoglycemia and prematurity, could result in behavioral problems and developmental delay [5,6,7]. More recently, impairments in the areas of emotional-behavioral functioning and social relationships, assessed through a parent-compiled questionnaire, were described in 87 children with BWS [8]. However, the study of Kent and colleagues did not consider any developmental difficulties presented by children with BWS, which could affect their emotional-behavioral and social functioning.

Even though the prognosis is essentially favorable, BWS can be considered as a chronic illness since it is a life-long condition that requires ongoing medical attention [4,9]. Especially in the first years of life, children with BWS are frequently subjected to invasive diagnostic procedures and surgical interventions (e.g., tongue reduction) [10]. As in other chronic pediatric diseases, in BWS, frequent hospitalizations, restrictions in daily activity and concerns about physical appearance might increase the risk for emotional-behavioral difficulties [11] and affect diverse areas of development, such as motor abilities, language acquisition and social adjustment [12,13]. Accordingly, sequelae on psychosocial functioning in the first years of life have been reported for some main features of BWS, namely, macroglossia [14] and abdominal wall defects [15,16]. These findings suggest that, even in absence of a diagnosis of neurodevelopmental disorder, preschool children with BWS may present psychosocial difficulties. Nevertheless, it is still lacking a detailed investigation of emotional-behavioral difficulties and of psychomotor and social development in this population.

Examining these aspects would be particularly important in preschool age. This stage of development represents, indeed, a critical period for identifying possible at-risk conditions that have not yet become structured and eventually programming psychological interventions and supports [17]. Moreover, in this development phase, parents are privileged observers with respect to later periods of growth, so that questionnaires and checklist could be considered as reliable instruments to assess behavioral difficulties and specific areas of child development [18].

In the light of these premises, the current exploratory study investigated the presence of psychosocial difficulties in preschool children with BWS without documented neurological and psychiatric diseases. Parents were asked to fill out two standardized questionnaires assessing emotional-behavioral problems and the developmental level in different domains, from social to motor skills, with the aim to describe the behavioral and developmental profile of BWS in preschool age.

## 2. Materials and Methods

### 2.1. Participants

Thirty participants were recruited in collaboration with the Italian Association of Beckwith–Wiedemann Syndrome (AIBWS). Inclusion criteria were: (i) confirmed clinical and/or genetic diagnosis of BWS, (ii) age > 1.5 years and < 6 years and (iii) absence of documented neurological and psychiatric conditions (e.g., epilepsy, autism spectrum disorder). This latter criterion allowed us to verify whether preschool children with BWS presented psychosocial difficulties that were not secondary to the presence of a neurodevelopmental disorders. In total, 7 participants were excluded, corresponding to 19% of the sample. This percentage is in line with recent literature documenting the prevalence of neurodevelopmental disorders in children younger than eight years old in the USA [19]. 

Recruitment was country-wide, and was conducted in two different time windows (2012–2013, 2016–2017).

### 2.2. Procedure

The families enrolled in the AIBWS received a letter from the president of the Association informing them of the possibility of participating to the study. All interested families were then sent an envelope containing: (a) an informed consent form; (b) an ad-hoc information form to collect socio-demographic and clinical variables; and (c) the two questionnaires assessing emotional-behavioral problems and different developmental areas. Parents were asked to sign the informed consent form and fulfill all the documents before sending them back via mail. All procedures of the study were in accordance with the Declaration of Helsinki and were approved by the Ethical Committee of the Scientific Institute, IRCCS E. Medea. Please note that the study was carried out before the COVID-19 pandemic.

### 2.3. Behavioral and Emotional Problems 

Parents filled out the Child Behavior Checklist (CBCL 1.5–5), an internationally adopted, standardized questionnaire designed to assess various types of behavioral and emotional problems in children aged 1.5 to 5 years [18]. The CBCL 1.5–5 provides the following 7 syndrome scales: Emotional Reactivity; Anxiety/Depression; Somatic Complaints; Withdrawal; Sleep Problems; Attention Problems; and Aggressive Behaviors. Raw scores of each scale were summed up and then transformed into T-scores (mean = 50, SD = 10) according to the normative values, so as a higher score indicated higher behavioral problems in that scale. Moreover, the CBCL 1.5–5 provides cutoff scores according to percentile distribution so as to determine children scoring in the borderline and in the clinical range. The term clinical is used here as being synonymous with problematic, thus referring to children who show consistent problems in their behavior, without any psychopathological evaluation of these problems having been made.

### 2.4. Child’s Development 

The child’s development was assessed using the Child Development Inventory (CDI [20]), a parent-report questionnaire that describes children’s abilities from 15 months to 6 years of age. To obtain a profile of the child’s development, the items are summed up into the following scales: Social development; Self-help; Gross-motor; Fine-motor; Expressive language; Language comprehension; Letters knowledge; and Numbers knowledge. Raw scores obtained by summing the items of each scale were converted into T-scores according to the mean expected for each age group reported in the original manual. This way, the lower was the T-score, the lower the developmental level was in that scale. According to the normative manual, scores ≤1.5 SD and ≤2 SD were considered, respectively, as falling within the borderline and the clinical range. Similarly to the CBCL 1.5–5, the term clinical adopted here does not reflect a diagnosis of developmental delay; rather, it helps to identify those children whose development is questionable and who could show less expected age-related competences in each specific area.

### 2.5. Socio-Economic Status (SES) 

SES was coded according to the information provided by caregivers on the basis of Hollingshead’s [21] classification for parental occupation. Scores ranging from 70 to 90 correspond to the upper status, while scores ranging from 40 to 65 correspond to the middle status and scores ranging from 10 to 35 correspond to the lower status.

### 2.6. Statistical Analyses

Preliminarily, descriptive statistics and the percentage of children exceeding the borderline and clinical thresholds were calculated for each scale of the two questionnaires. For the scales in which the number of children exceeding the borderline threshold was >20%, we adopted chi-squared tests among dummy variables of the two questionnaires to verify whether the same individuals had behavioral problems and difficulties in specific developmental domains.

Then, for each scale of the two questionnaires, we ran Spearman’s r correlations and Student’s t-tests with selected, background continuous variables and categorical factors, respectively. Specifically, to control for socio-demographic variables, we inserted gender, age and SES into analyses. In line with previous literature [1,14,15], we also considered clinical variables that have been pointed as risk-factors for psychosocial development, namely, prematurity, neonatal hypoglycemia, abdominal wall defects and macroglossia, and the clinical score obtained by each child according to the Consensus statement [2].

For each test, a false-discovery rate analysis (FDR) was conducted to control for multiple testing, thus correcting the accepted *p*-value according to the number of comparisons [22]. Eventually, significant background variables were inserted as covariates into repeated-measure analyses of covariance (ANCOVAs) separately for the two questionnaires, with scale as within-subject variable. Significant interaction effects were further examined with Tukey HSD post-hoc tests. The α value was set at *p* < 0.05 for all statistical tests. Effect sizes for the ANCOVAs were reported as partial Eta squared (η2p), adopting conventional cut-offs of η2p = 0.01, 0.06 and 0.14 for small, medium and large effect sizes, respectively [23]. Data were reported as mean and standard error of the mean (SEM). All analyses were performed by means of the Statistica software version 8 (Statsoft, Tulsa, OK, USA).

## 3. Results

### 3.1. Socio-Demograsphic and Clinical Variables

A description of the socio-demographic and clinical variables of the sample is reported in Table 1.

For the CBCL 1.5–5, significant correlations emerged between age and both the Emotional reactivity (r = 0.45, *p* = 0.012) and Anxiety/Depression scales (r = 0.61, *p* < 0.001), while all other findings for either continuous or categorical variables were non-significant (all r < |0.39|, all t < 2.65, all *p* ≥ 0.013). 

In a similar vein, for the CDI, age was significantly correlated with the Social development (r = −0.79, *p* < 0.001), Self-help (r = −0.45, *p* = 0.013), Gross-motor (r = −0.45, *p* = 0.014) and Letters knowledge scales (r = −0.47, *p* = 0.009). Moreover, a significant association emerged between familial SES and the Numbers knowledge scale (r = 0.53, *p* = 0.003), while all other correlations and t-test analyses were non-significant after controlling for multiple testing (all r < |0.42|, all t < 2.26, all *p* > 0.020).

### 3.2. ANCOVA

**Figure 1:**
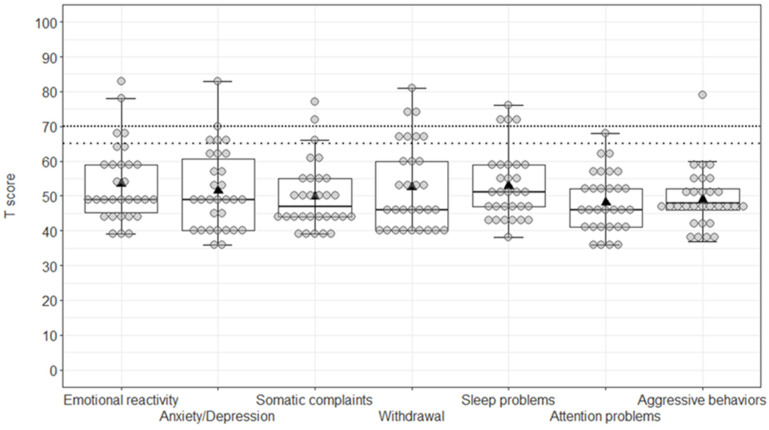
Boxplot of T-scores at the CBCL/1.5–5. Grey circles (●) represent individual scores, black triangles (▲) indicate group mean scores; lines with wide and dense dots show, respectively, the borderline and clinical thresholds.

For the CBCL 1.5–5, the ANCOVA confirmed the significant effect of the covariate age (F1,28 = 9.98, *p* < 0.001, η2p = 0.26), indicating that the older the age was, the higher the obtained scores were at the CBCL/1.5–5 (r = 0.51, *p* = 0.004). All other effects were non-significant (all F < 1.62, all *p* > 0.144), thus highlighting no differences between the scales (Figure 1).

**Figure 2:**
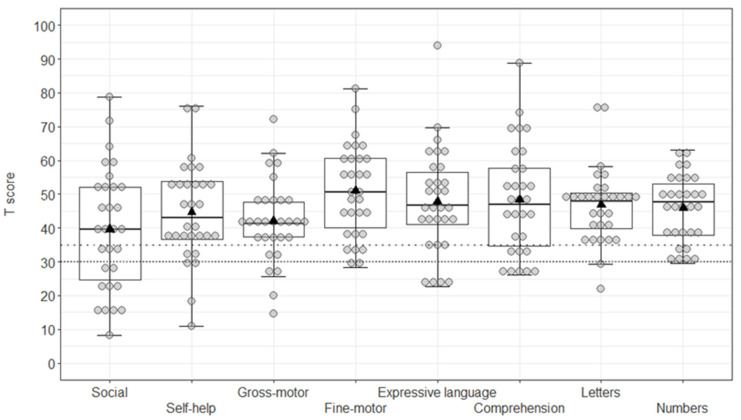
Boxplot of T-scores at the CDI. Grey circles (●) represent individual scores, black triangles (▲) indicate group mean scores; lines with wide and dense dots show, respectively, the borderline and clinical thresholds.

For the CDI, the ANCOVA confirmed a significant age effect (F1,27 = 17.22, *p* < 0.001, η2p = 0.39), with a decrease in T-scores in older children across the scales (r = −0.64, *p* < 0.001). Furthermore, the interaction scale × age was significant (F7,189 = 3.32, *p* = 0.002, η2p = 0.11). The Tukey HSD post-hoc comparisons indicated lower scores at the Social development scale than at the Fine-motor (*p* = 0.001) and Language comprehension (*p* = 0.036) scales. Moreover, lower T-scores were reliable at the Gross-motor compared to the Fine-motor scale (*p* = 0.026). All other effects were non-significant (all F < 1.191, all *p* > 0.178) (Figure 2).

### 3.3. Associations between the Two Questionnaires

As regards the possible associations between behavioral problems and specific developmental difficulties, the chi-squared tests did not highlight significant results (all chi-squared < 0.72, all *p* > 0.398).

## 4. Discussion

In the current study, we examined the presence of emotional-behavioral problems and of difficulties in specific developmental domains, from motor to social functioning, in preschool children with BWS through two standardized parent-report questionnaires. The results indicated that overall BWS was not associated with specific behavioral problems, but, at the individual level, almost a quarter of the children in the sample presented scores beyond the borderline threshold for the Withdrawal scale. As concerns specific areas of development, a lower group mean score emerged in the Social development scale compared to others, and almost half of the sample obtained individual scores within the borderline or the clinical range in the social domain. Lower scores also emerged for the Gross-motor compared to the Fine-motor scale. Moreover, in the Gross-motor and Language comprehension scales 23% and 27% of the sample presented scores within the borderline or clinical bands, respectively. Of note, older ages were associated with higher behavioral problems and lower developmental scores across the scales of both the questionnaires, while no other socio-demographic or clinical variables accounted for the scores obtained in the two questionnaires. 

Partially in contrast with the study of Kent and colleagues [8], our results regarding the emotional-behavioral problems highlighted neither a group score lower than the expected mean nor significant differences between the scales. This inconsistency might depend on the age range of the samples, since we limited them to preschool children, while Kent and colleagues recruited children from preschool age to adolescence. On the other hand, as also shown by our results, higher behavioral problems could arise as age increases.

Moreover, almost 7% of the children in the study of Kent and colleagues had a diagnosis of autism while, here, the presence of documented neuropsychiatric diagnosis was considered as an exclusion criterion. Nevertheless, when we look at the individual performance, 7 out of 30 children presented problems of social withdrawal. Previous research documented that children with different chronic diseases tend to show less prosocial behavior and could present emotional problems such as anxiety and depression symptoms [12,24]. Interestingly, here, we also found that increasing age was associated with higher emotional reactivity and anxiety/depression problems. Overall, despite the fact that these results do not highlight a specific behavioral profile, they suggest that, even in the absence of neurodevelopmental disorders, preschool children with BWS could present problems in their emotional experience and in participating in the social context, and these difficulties could increase in older ages.

Regarding the psychomotor and social development, the results highlighted reliable differences between the scales, with developmental difficulties in the social domain, which became more pronounced in older children. Moreover, the 43% of children obtained scores exceeding the borderline threshold for social development, with even 10 out of 30 children scoring within the clinical range. Thus, according to previous findings suggesting that children with chronic illness exhibit difficulties in social interaction [25,26], our study corroborated that, already at preschool age, children with BWS showed reduced interpersonal skills, which could become more pronounced in older children [8].

It is worth noting that out of seven children with withdrawal problems, four had scores in the borderline (N = 1) or clinical (N = 3) ranges for the Social development scale, two were in the borderline (N = 1) or clinical (N = 1) ranges for the Gross-motor scale and one fell in the borderline range for the Language comprehension scale. The analyses, however, indicate that social withdrawal problems were independent from developmental difficulties in the social domain or in other scales. This suggests that despite problems of withdrawal and delays in acquiring age-appropriate social skills potentially affecting the social functioning of children with BWS, it is quite possible that there is not a direct association between these variables. As an example, a child may have adequate social skills but appear as withdrawn and, vice versa, he/she could not show problems of social withdraw despite having fewer social competences compared to peers. As a consequence, our findings highlight that both these aspects are worthy to be monitored by caregivers, clinicians, and educational professionals.

For the CDI, a significant difference was also reliable between gross-motor and fine-motor skills, with lower scores obtained at the former scale. This result might depend by overgrowth conditions typical of the syndrome [1], which would mainly affect gross-motor abilities, such as walking, running or climbing. This discrepancy, however, should be taken into account for screening and assessment in the first years of life, even considering that 7 out of 30 children scored beyond the borderline threshold. Moreover, for the Language comprehension scale, a high percentage (27%) of children were in the borderline (N = 3) or in the clinical range (N = 5). Given the critical importance of comprehension abilities in the preschool period for the general cognitive functioning [27], it would be useful to monitor difficulties in this area during routine pediatric evaluation.

Importantly, increasing age was overall associated with higher behavioral and developmental difficulties. Previous research on children with typical development documented that, across diverse countries and cultures, problems in emotional reactivity, social withdrawn, anxiety and depression increase with age [28]. In a similar vein, a study regarding another pediatric rare disease, that is congenital central hypoventilation syndrome, reported that problems in diverse areas of development, and particularly social functioning, were reliable across different age groups with the exception of children younger than 3 years old [29]. In this light, we would speculate that children with BWS might become more aware of their condition as age increases and also because they spend more time in social contexts outside the family so that they could experience being different from peers [12,24,25]. In line with this speculation, it would be helpful to monitor emotional and psychosocial difficulties of children with BWS when entering at the kindergarten and, later, at school [30].

Notably, no other socio-demographic and clinical variables were associated with emotional-behavioral and developmental problems. This finding suggests that, beyond the presence of risk factors such as prematurity or neonatal hypoglycemia, preschool-age children with BWS could present psychosocial difficulties, which might depend on their experience of living with a rare disorder that requires complex medical assistance since the first years of life.

The results of this study should be discussed considering several limitations. First, even though BWS is a rare syndrome, the sample size is relatively small and includes a higher number of female than male participants. Preliminary t-tests, however, did not highlight significant differences in both the questionnaires between boys and girls. Moreover, despite the adoption of validated, standardized questionnaires provides reliable results, the lack of an age-matched, control group asks for caution in generalizing our findings. While we controlled for possible effects of background demographic and clinical variables, our sample size prevented us from investigating the role of other familiar conditions and parental psychological variables as well as of each genotype. Since Paternal Uniparental Disomy was reported to be frequently associated with neurodevelopmental problems [3,8], future studies on wider samples should investigate whether specific (epi)genotype-phenotype could be associated with behavioral and developmental problems [31]. Lastly, we decided to include children without neurodevelopmental disorders, a criterion that could have biased our sample. On the other hand, this choice ensured us that the social and emotional-behavioral difficulties reported here were not secondary to other neurological or psychiatric conditions.

Despite these limitations, this study provides first evidence that preschool-age children with BWS could present psychosocial difficulties, sustaining that standardized assessments of these aspects should be included in routine follow-up evaluations, even when there are no previous diagnoses of neurological or psychiatric disorders [10]. This way, it would be possible to detect children that require rehabilitative/educational interventions and psychological support early before possible emotional-behavioral disorders become structured [17]. An early psychological assessment would also have potential beneficial outcomes for the national health system, as it would reduce the costs associated with long-term consequences of neglected emotional-behavioral problems [11]. In sum, even if further research on BWS is required, this study would be a first step for a further consideration of the psychosocial sequelae associated with this rare syndrome.

## Figures and Tables

**Table 1:** Socio-demographic and clinical variables of the sample.

	Mean (SD)/N (%)	Notes
Demographic variables		
Sex (males)	8 (27%)	
Age (years)	3.3 (1.4)	
Familiar variables		
Maternal age (years)	37.7 (4.6)	
Maternal education (years)	13.7 (3.3)	
Paternal age (years)	41.2 (5.8)	
Paternal education (years)	13.3 (3.2)	
Socio-economic status	57 (19)	Corresponding to a medium–high level according to Hollingshead (1975)
Siblings	0.9 (0.7)	
Perinatal variables		
Birth Weight (g)	3427 (643)	
Birth Length (cm)	51 (4)	
Prematurity	13 (43%)	13 moderate-to-late preterm (32 to 37 weeks)
Genetic diagnosis		
Altered expression of *IGF2*	2 (7%)	
Altered expression of *CDKN1C*	21 (70%)	
Paternal Uniparental Disomy	5 (16%)	
Other	2 (7%)	1 altered methylation of both IC1 and IC2, 1 unknown
Main clinical features		
Macroglossia	24 (80%)	
Omphalocele /abdominal wall defects	12 (40%)	
Birthweight/Length > 2 ds above the mean	10 (33%)	
Neonatal hypoglycemia	10 (33%)	
Lateralized overgrowth	13 (43%)	
Tumor onset	1 (3%)	1 hemangioendothelioma
Clinical index according to the Consensus statement (2018)	5.1 (1.8)	

*IGF2*: Insulin Like Growth Factor 2; *CDKN1C*: Cyclin Dependent Kinase Inhibitor 1C; IC1: Imprinting Center 1; IC2: Imprinting Center 2.

## Data Availability

Data related to this study will be made available upon reasonable request by sending an email to the corresponding author.
